# Ecological drivers of marine debris ingestion in Procellariiform Seabirds

**DOI:** 10.1038/s41598-018-37324-w

**Published:** 2019-01-29

**Authors:** Lauren Roman, Elizabeth Bell, Chris Wilcox, Britta Denise Hardesty, Mark Hindell

**Affiliations:** 1 Oceans and Atmosphere, Commonwealth Scientific and Industrial Research Organisation, Hobart, Tasmania Australia; 2 Institute for Marine and Antarctic Studies, University of Tasmania, Hobart, Australia; 3 Wildlife Management International, Blenheim, New Zealand; 4 Antarctic Climate and Ecosystems CRC, University of Tasmania, Hobart, Tasmania Australia

## Abstract

Procellariiform seabirds are both the most threatened bird group globally, and the group with the highest incidence of marine debris ingestion. We examined the incidence and ecological factors associated with marine debris ingestion in Procellariiformes by examining seabirds collected at a global seabird hotspot, the Australasian - Southern Ocean boundary. We examined marine debris ingestion trends in 1734 individuals of 51 Procellariform species, finding significant variation in the incidence of marine debris abundance among species. Variation in the incidence of marine debris ingestion between species was influenced by the taxonomy, foraging ecology, diet, and foraging range overlaps with oceanic regions polluted with marine debris. Among the ecological drivers of marine debris ingestion variability in Procellariiformes, we demonstrate that the combination of taxonomy, foraging method, diet, and exposure to marine debris are the most important determinants of incidence of ingestion. We use these results to develop a global forecast for Procellariiform taxa at the risk of highest incidence of marine debris ingestion. We find seabirds that forage at the surface; especially by surface seizing, diving and filtering, those with a crustacean dominant diet, and those that forage in or near marine debris hotspots are at highest risk of debris ingestion. We predict that family with the highest risk are the storm petrels (*Hydrobatidae* and *Oceanitidae*). We demonstrate that the greater the exposure of high-risk groups to marine debris while foraging, the greater the incidence and number of marine debris items will be ingested.

## Introduction

Ingestion of plastics and other debris in the marine environment is a widespread, emerging threat to seabirds^1,2^, which mistake plastics for food^3^. Presently, 50% of the world’s seabird species have been reported to be affected by marine debris ingestion^4^. With an estimated 15–51 trillion plastic pieces currently floating in the world’s oceans^5^, and more entering daily, floating plastics and other marine debris pose a growing risk to seabirds^2^.

Seabirds predicted to be at greatest risk are those within the Southern Ocean boundary, particularly surrounding the Tasman sea between Australia and New Zealand, as it is an identified hotspot for risk of debris ingestion in seabirds^6^. Multi-species studies investigating plastic consumption in aquatic and marine birds report that Procellariiformes ingest marine debris at greater frequency than the eight other avifauna orders studied^7,8^. There are 90 species of Procellariiform seabird in the Oceania region surrounding the Southern Ocean and Tasman sea boundary, of which 37 species are threatened (listed as vulnerable, endangered, or critically endangered on the IUCN red list^9^), and 14 are listed as near threatened. Fifty four percent of these 90 Procellariiform species are in population decline due to a variety of threats^9^.

The rates of plastic ingestion vary substantially between Procellariiform species. Previous studies show that *Diomedea* and *Thalassarche* albatrosses rarely ingest plastics^10^, while plastic ingestion rates exceed 90% in other species, including Cory’s shearwater, *Calonectris diomed*ea^11^, Northern fulmar*, Fulmarus glacialis*^12^, and short-tailed shearwater, *Ardenna tenuirostris*^8^. Data explicitly examining plastic ingestion in seabirds globally is patchy, with comprehensive data available for just a small proportion of common or accessible species^13^.

Recent modelling of plastic ingestion in all seabird species, including Procellariformes, using empirical data suggests that the incidence of debris ingestion in seabirds increases with increasing exposure^6^, geography^10^ and foraging behaviour^14^; with higher debris ingestion in species with zooplankton diets and surface feeding^8^. However, there has yet to be a synoptic, comprehensive, multi-species study of Procellariiformes examining the relative contribution of potential drivers which may put some species at greater risk of marine debris ingestion than others. In this study, we add to this previous modelling^6^ by evaluating the relative importance of ecological drivers of debris ingestion in Procellariiform seabirds and used this information to forecast which seabird groups are at the highest risk of debris ingestion. Using the incidence of debris ingestion across 51 Procellariiform species, we determined a set of ecological criteria useful for predicting risk of marine debris ingestion.

## Results

### Incidence and magnitude of marine debris ingestion

We collected and necropsied 1734 individual adult and immature seabirds of 51 species, representing all four families of Procellariiform seabirds; *Diomedeidae*, *Procellariidae*, *Hydrobatidae* and *Pelecanoididae*. All four families studied ingested debris (SI Table 1). Overall, debris was recorded in 32% of individuals and 31 of 51 species examined, with the highest number of items ingested by an individual bird being 40 items. Among individuals that had ingested debris, a mean 4.95 and a median of 3 items were ingested (SI Table 1).

### Ecological drivers of marine debris ingestion in Procellariiformes

When single ecological factors are examined in isolation, species is the best single predictor of number of ingested marine debris items, with 50.1% of deviance explained (R^2^ = 0.281).

**Figure 1:**
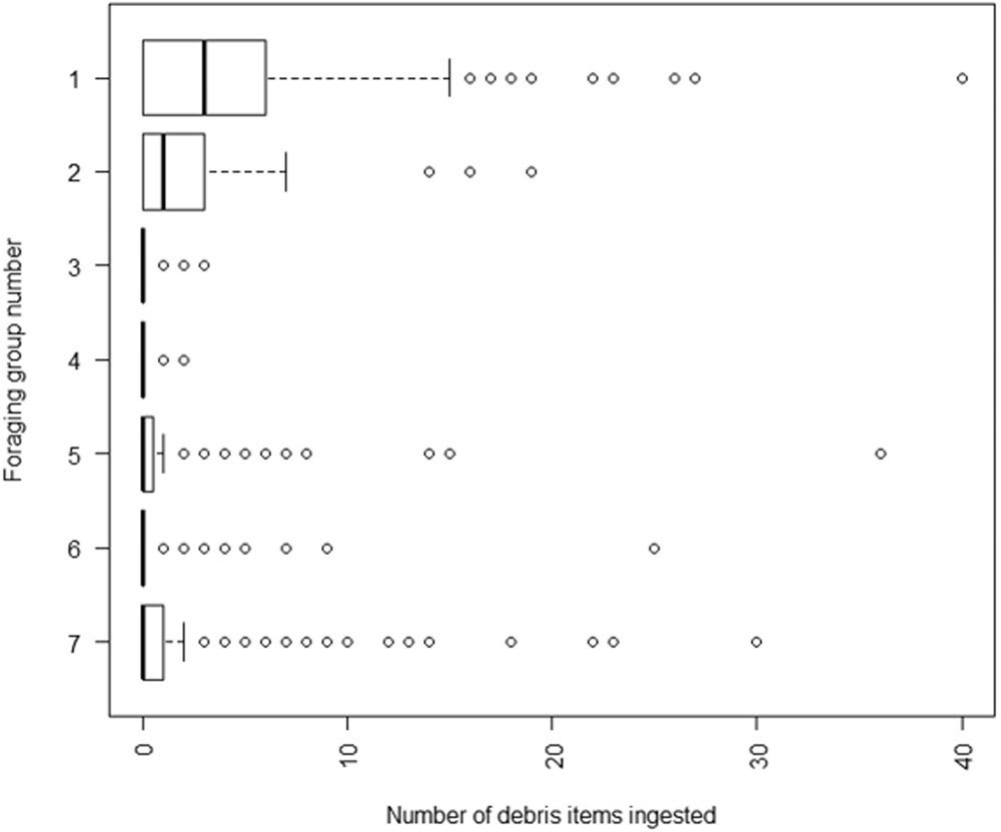
Box-plot of number of marine debris ingested by seven foraging groups determined by cluster analysis of seabird foraging behaviour following Marchant and Higgins (1990). Group 1: mostly surface diving and pursuit diving/plunging. Group 2: mixed surface foraging including filtering, also seizing, plunging and dipping. Group 3: feeding under the surface by pursuit diving/plunging. Group 4: surface seizing and diving. Group 5: mostly pursuit plunging with some surface and pursuit diving. Group 6: surface seizing and plunging with minimal other feeding methods. Group 7: surface seizing, pattering and dipping. This area bounded by each box within the plot area shows the interquartile range (IQR) between the first quartile (left edge of box) and third quartile (right edge of box). The bold line in the middle of the box shows the median. The whisker shows 1.5x IQR. Each circle represents outlier values.

A cluster analysis of foraging behavior grouped the species into seven foraging clusters (Log-likelihood = −4571.6). Foraging groups can be broadly described as: mostly surface diving and pursuit diving/plunging (group 1), mixed surface foraging including filtering, seizing, plunging and dipping (group 2), feeding under the surface by pursuit diving/plunging (group 3), surface seizing and diving (group 4), mostly pursuit plunging with some surface and pursuit diving (group 5), surface seizing and plunging with minimal other feeding methods (group 6) and surface seizing, pattering and dipping (group 7). Foraging method explained 32.6% of the deviance (R^2^ = 0.187) and was the next single best predictor. Foraging groups 1, 2, 3, 4 and 7 had significant variation in the number of ingested debris items. While all foraging groups ingested marine debris, groups 1 and 2 had greater ingestion compared to other foraging groups (Fig. 1).

**Figure 2:**
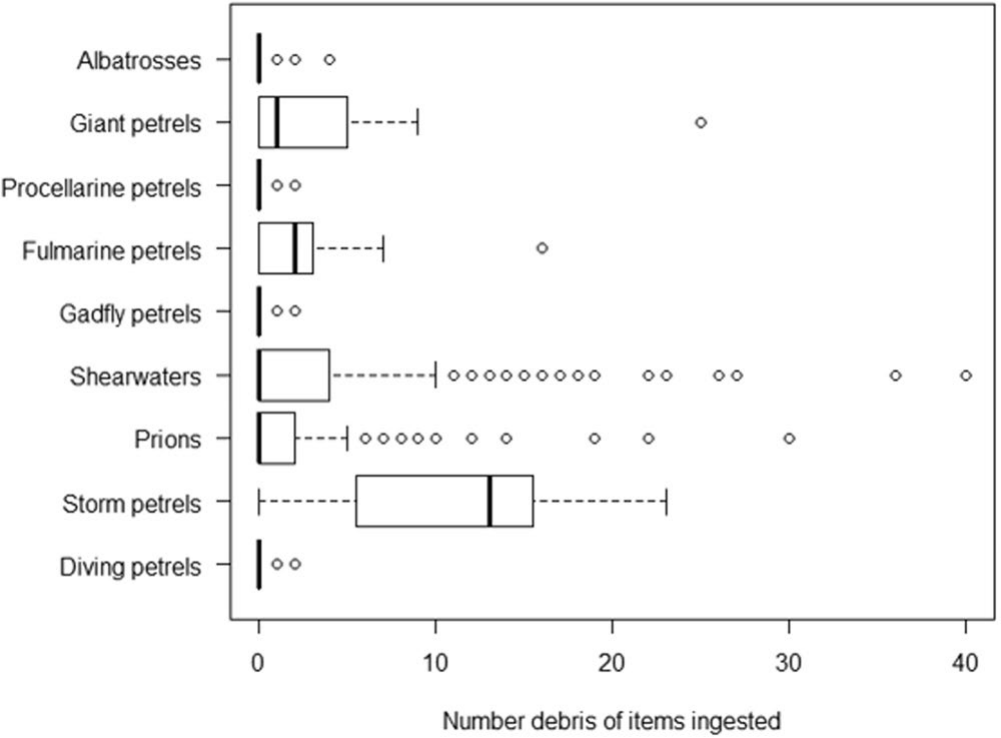
Box-plot of number of marine debris ingested by Procellariiform taxonomic groupings. This area bounded by each box within the plot area shows the interquartile range (IQR) between the first quartile (left edge of box) and third quartile (right edge of box). The bold line in the middle of the box shows the median. The whisker shows 1.5x IQR. Each circle represents outlier values.

Taxonomic grouping significantly influences the number of items ingested by a seabird for albatrosses, fulmarine, gadfly and giant petrels, shearwaters, prions and storm petrels, explaining 31.7% deviance in number of items ingested (R^2^ = 0.129) (Fig. 2). Storm-petrels ingested the most marine debris (median = 13, standard deviation = 8.1), followed by fulmarine petrels (median = 2, standard deviation = 4.6), and giant petrels (median = 1, standard deviation = 7.5). Procellarine petrels (median = 0, standard deviation = 0.2) ingested the least marine debris, followed by diving petrels (median = 0, standard deviation = 0.4) (Fig. 2).

**Figure 3:**
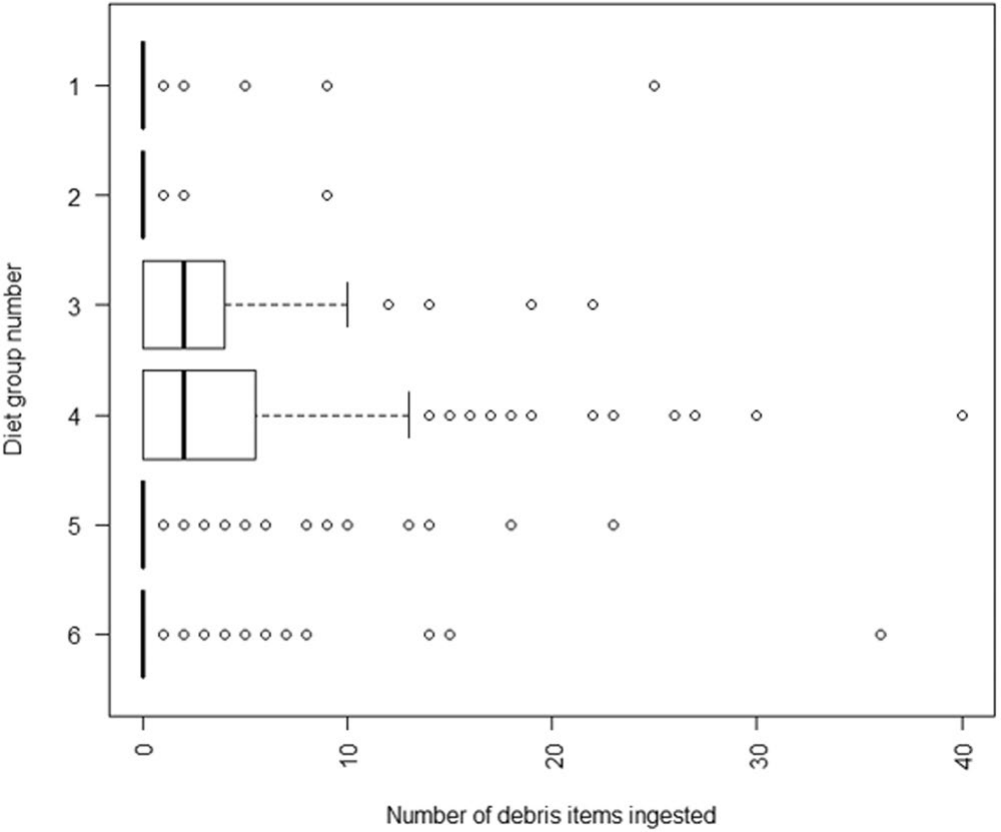
Box-plot of number of marine debris ingested by six diet groups determined by cluster analysis of seabird primary diets following Marchant and Higgins (1990). Group 1: diet squid dominant, with some fish, crustaceans and scavenging. Group 2: diet fish dominant, but some squid and crustaceans. Group 3: diet crustacean dominant but may take fish and squid. Group 4: diet cephalopods and crustaceans dominant, with some also taking fish. Group 5: diet mostly fish and crustaceans. Group 6: diet mostly fish and squid with some scavenging. This area bounded by each box within the plot area shows the interquartile range (IQR) between the first quartile (left edge of box) and third quartile (right edge of box). The bold line in the middle of the box shows the median. The whisker shows 1.5x IQR. Each circle represents outlier values.

Cluster analysis sorted birds’ diets into six diet clusters (Log-likelihood = −3641.9). Diet groupings can be generally described as: squid dominant, with some fish, crustaceans and scavenging (group 1), fish dominant, but some squid and crustaceans (group 2), crustacean dominant but may take fish and squid (group 3), cephalopods and crustaceans dominant, with some also taking fish (group 4), mostly fish and crustaceans (group 5), and mostly fish and squid with some scavenging (group 6). Diet grouping explained 30.8% of deviance in debris ingested (R^2^ = 0.154), and diet groups 1, 3, 4, 5 and 6 were significantly correlated with marine debris ingestion. While all diet groups ingested marine debris, diet groups 3 (median = 2, standard deviation = 3.67) and 4 (median = 2, standard deviation = 5.13) ingested the highest number of items (Fig. 3).

Encounter density was significantly positively correlated with the number of debris items ingested by a seabird, explaining 11.4% of deviance in ingested items (R^2^ = 0.154). The deviance explained by encounter density did not overlap with the deviance explained by diet and foraging method.

### The best model to predict marine debris ingestion in Procellariiform seabirds

The best model to explain the sum of debris items ingested by a seabird includes taxonomic group, diet group, foraging group and encounter density (df 22, log likelihood −4199.65, AIC 8443.9), explaining 48.9% of deviance. Species was excluded from this model to prevent overfitting. A model including the presence/absence of an isthmus juncture in the gastrointestinal tract is equivalent to this best model (df 22, log likelihood −4199.65, AIC 8443.9).

### Case studies

1. Fluttering shearwaters, *Puffinus gavia*,  and fairy prions, *Pachyptila turtur*, inhabit a large overlapping range in the Tasman sea^15^. Both fluttering shearwater and fairy prion were allocated diet group 5 (diet mostly fish and crustaceans) by dietary cluster analysis. Fluttering shearwater was allocated foraging group 3 (feeding under the surface by pursuit diving/plunging) and fairy prion was allocated foraging group 7 (surface seizing, pattering and dipping) by foraging strategy cluster analysis. An ANOVA demonstrates that fluttering shearwater (n = 70, mean debris ingested = 0.16 ± 0.5 items) ingest significantly less (P = 0.02) marine debris than fairy prions (n = 236, mean debris ingested = 0.63 ± 1.6 items).
2. Flesh-footed shearwater, *Ardenna carneipes,* foraging range was split at longitude 145 into an eastern and western foraging population. The eastern population was significantly (AIC = 223.83, P < 0.01) more likely to ingest marine debris, with 59.4% debris ingestion, than the western population with 18.75% debris ingestion. There is a significant difference (R^2^ = 0.158, P < 0.01) in the number of items ingested by flesh-footed shearwaters from eastern (median = 2, standard deviation = 6.94) and western populations (median = 0, standard deviation = 1.06) (Fig. 4). The ratio of expected use of foraging habitat, as per Wilcox (2015) east of longitude 145 to west of longitude 145 is 0.57:1. After accounting for expected use, the adjusted encounter density ratio for the eastern and western population is 1:0.501.

**Figure 4:**
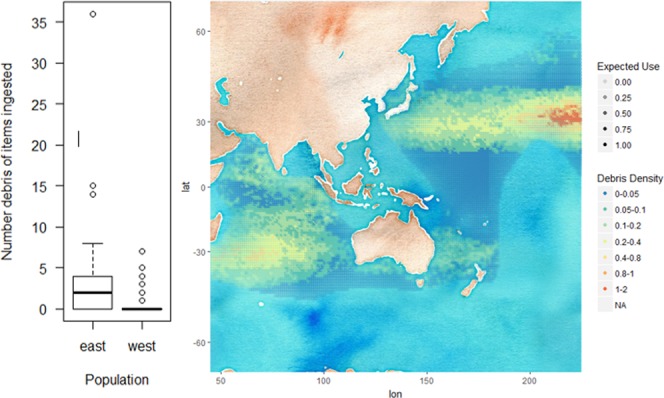
Box-plot (left) of number of marine debris ingested by flesh-footed shearwater, *Ardenna carneipes*, populations foraging to the east of longitude 145, and west of longitude 145. Map (right) of density of marine debris within the foraging range of flesh-footed shearwaters.
3. A subset of short-tailed shearwaters (n = 201) were collected during a 2013 November and December wreck during their return migration. Birds sampled from this wreck had ingested an average of 4.43 ( ± 4.17) items.

## Discussion

Debris ingestion by an individual seabird can be predicted by its foraging strategy, taxonomic grouping, diet and the environmental exposure to marine debris pollution, demonstrating that the ecological drivers of marine debris ingestion are complex and cannot be attributed to a single variable. Understanding the relative contributions of these ecological factors to the incidence of marine debris ingestion is useful for forecasting seabird species at high risk of marine debris ingestion – and the geographic areas of highest risk. Taxonomy (e.g. species) is the best single predictor variable of marine debris ingestion, with species capturing over half of all variation in debris ingestion in this study, suggesting that incidence of debris ingestion within a species can be predicted if incidence is known in a subset of that species. Though taxonomy itself is not strictly an ecological factor, it is included in this study as a proxy for shared behaviours within a related group of birds.

Though debris ingestion occurred across all foraging groups, those that forage below the sea surface and by pursuit of prey (groups 3 and 4) were at the lowest risk of ingesting marine debris. This may be due to buoyant marine debris occurring mostly at the ocean’s surface, and passively floating debris may not trigger the instinct of a bird which forages by pursuit. Mixed surface feeding strategies were associated with higher levels of debris ingestion, with the greatest risk of high debris loads associated with species that employ surface seizing, diving and filtering foraging strategies, as has been observed in previous studies comparing filter feeding and surface seizing to prey pursuing seabirds^16–18^. In our first case study, a significantly lower sum of debris was observed in the diving (group 3) fluttering shearwater than the surface feeding fairy prion (group 7), with both species inhabiting an overlapping range and exhibiting a similar diet, reinforces the observation of higher debris ingestion in surface feeding birds. The higher risk associated with surface filtering may result from non-food debris filtered from the water along with food during foraging bouts.

Debris ingestion occurred within all diet groups but was most abundant in seabirds with crustacean dominant diets, and least common in birds having fish dominant diets. This demonstrates that crustacean dominant diets are a major risk factor for marine debris. It’s possible that there is a dietary resemblance between some small crustaceans and hard plastic marine debris^8^, but plastic ingestion while feeding on crustaceans may be accidental as birds feed on pelagic crustaceans floating among plastic. In contrast, floating plastic and other debris does not typically resemble fish either by shape or behavior, and this is probably why marine debris is not attractive to piscivorous seabirds. These findings reflect previous observations of plastic ingestion occurring more commonly among seabirds with crustacean diets and less commonly among predominantly piscivorous seabirds^17^.

Encounter density is an important driver of marine debris ingestion, explaining variation in debris ingestion incidence that was not explained by diet, foraging method nor taxa (SI Table 2). The effect of exposure of Procellariiform taxa to regions heavily polluted with marine debris is clear when comparing debris ingestion in polluted and unpolluted regions. In the heavily polluted Hawaiian Islands region, seabirds ingest debris at much higher rates than birds from our study. The majority of Hawaiian albatrosses, including Laysan (89.5%) and black-footed albatrosses (58.8%) and gadfly petrels; including Bonin petrel (100%)^19^, ingest marine debris, while Australasian albatrosses and gadfly petrels displayed very low incidences of debris ingestion (Fig. 2). White-chinned petrels have only 0.9% incidence of debris in Australasia compared to 63.1% off Brazil, a region with much higher rates of plastic pollution^20^. Other studies also highlight environmental exposure to debris as an important factor in incidence of debris ingestion^21^. Van Franeker and Law (2015) highlight that fulmarine petrels that forage exclusively within relatively unpolluted Antarctic seasonal sea ice zone do not ingest plastic, but those that winter outside this region ingest plastic on these trips^21^. As many seabirds have a broad distribution and exact foraging routes of individuals are not known, the encounter density variable represents a range average and cannot capture local spatial or temporal effects. While encounter density provides an average snapshot of encountered marine debris in a species range, it does not account for specific breeding/wintering distributions, temporal offload of debris during chick rearing, nor trends of individual birds which forage in areas of locally high or low marine debris. Modelling accuracy could be improved with the inclusion of seabird population specific tracking data, local background debris availability data, and better representation of species with small sample sizes.

In general, closely related species share common foraging strategies and diets (SI Table 1)^22^, and we propose that taxonomic grouping captures much of the diet and foraging information for many species. Storm petrels were the taxonomic group in this study with the highest incidence of marine debris ingestion (85.7%), and highest mean number of items ingested (mean 11.14 items, range 0–23). Though their diet and foraging groupings support a moderate risk of debris ingestion, storm petrels forage exclusively at the surface and have a crustacean-dominant diet, two high-risk factors for debris ingestion. High incidences of debris ingestion in storm petrels have been recorded in the literature in multiple ocean basins, including 100% of Tristram’s Storm-petrel, *Oceanodroma tristrami*, chicks collected in Hawaiian islands^23^ and 79% of white-faced storm-petrel remains in gull pellets collected in the North Atlantic^24^. A high debris encounter density likely also drives this high debris ingestion incidence pattern in storm petrels, though there may be additional unknown factors. With this evidence of high debris ingestion in multiple storm petrel species across multiple oceans, storm petrels are one of the taxonomic groups at very high risk of debris ingestion globally.

Giant petrels presented the second highest incidence (58.3%), and mean of ingested items (mean 4.18 items, range 0–25) of debris in this study, though placed in low-moderate risk foraging and diet groups and with low-moderate encounter density. Marine debris has been found in 72.7% of southern giant petrel pellets in Patagonia^25^. Giant petrels’ habit for scavenging and predating on smaller plastic-ingesting seabirds^25^ are a likely source of risk unique to this taxa. It is possible that the rates of plastic ingestion in giant petrels that we encountered, which are higher than expected from their ecological factors, are an artifact of giant petrels gaining plastic loads through secondary ingestion when scavenging other seabirds. In this study we encountered secondary ingestion of marine debris in a giant petrel that had eaten a shearwater with ingested plastic shortly before death, and giant petrel gut contents often contain seabird feathers (pers. obs.).

It is interesting to note that the AIC of the best model to explain the number of debris items ingested by a seabird was equivalent to a best model also including the presence/absence of a restricted isthmus juncture in the gut. The albatross taxonomic group is the only taxonomic group lacking this isthmus juncture, a gut structure that puts Procellariiformes at increased risk of retaining ingested plastic^26^. As albatross are the only bird group lacking an isthmus juncture, this is why isthmus juncture presence does not add to the best model as this information is already equivalently captured by the albatross bird group. The lack of isthmus juncture in albatrosses may also reduce detection of marine debris ingestion by albatrosses, compared to other taxonomic groups, if albatrosses regurgitate ingested marine debris before death.

Adding further to the body of evidence of the influence of environmental debris encounter to debris ingestion, here we provide a case study (2) of the effect of marine debris pollution in foraging ranges of two geographically populations of a single species, flesh-footed shearwater^27,28^, removed from the confounding effects of differing diet, foraging behavior and taxonomy. Flesh-footed shearwaters are widely distributed across the Indian and Pacific Oceans, and often solitary when foraging. Their movements cover a broad pelagic distribution, and individual flight paths vary considerably; with birds from different breeding colonies undertake markedly different migratory routes^27,28^. Tracks of individuals from the eastern Pacific Australasian flesh-footed shearwater populations show they migrate to the polluted north-west Pacific Ocean^27^. Tracking of the Indian (western) Australasian population shows that they migrate to the less polluted south-eastern Indian ocean^28^. The pattern of debris ingestion in eastern compared to western flesh-footed shearwaters reflects the debris they are likely to encounter in their respective foraging ranges, with significantly higher average debris ingestion in eastern (59.4% debris ingestion) than western (18.75% debris ingestion) birds (Fig. 4). The variability, including individuals with very high incidence of debris ingestion likely reflects the foraging tracks of individual birds into variably polluted regions of the Pacific Ocean.

Evidence for the influence of encounter of marine debris pollution in foraging ranges is also demonstrated by comparing species with known foraging paths or restricted ranges. Fluttering shearwaters feed in the relatively unpolluted Tasman Sea [15]. The average fluttering shearwater does not ingest marine debris, and the highest number of recorded items ingested was 3, demonstrating that debris ingestion is rare in species restricted to low pollution habitat. In contrast, short-tailed shearwaters follow an annual migration route passing through heavily polluted parts of the Northern Pacific Ocean during the Northern hemisphere summer and returning to the lesser polluted Southern Pacific and Southern Ocean during the Southern hemisphere summer [15]. The median short-tailed shearwater we collected ingested 4 debris items with a maximum of 27. In our third case study, short-tailed shearwaters collected the heavily polluted North Pacific Ocean in June-July (n = 87) contain a mean of 15.1 ± 2.9 marine debris items^29^, while those collected in the less polluted Southern Pacific Ocean in November-December for in this study, following their return migration (n = 201), contained a mean of 4.43 ± 4.17 marine debris items. Assuming marine debris concentrations in shearwater foraging habitat remain comparable between the studies undertaken in different years, this marked decline in ingested debris as this species migrated from heavily to marginally polluted foraging areas, adds to the evidence that exposure to marine debris is an important driver in debris ingestion.

Combining the results of ecological factors that contribute to increased debris ingestion in Procellariiform seabirds, we predict that storm petrels (*Hydrobatidae* and *Oceanitidae*) are the bird group currently at the greatest risk of a high incidence of debris ingestion due to satisfying this combination of high-risk ecological factors. We suggest that prions and fulmarine petrels display behavioral and dietary risk factors of high debris ingestion, though these bird groups largely inhabit the lesser polluted Southern Ocean. We predict that prions and fulmarine petrels are the groups most vulnerable to amplified debris ingestion if there is a future increase of plastic pollution in the Southern Ocean. We predict increased incidences of debris ingestion, and resulting harm, would occur across all Procellariiform species should their current level of environmental exposure to debris increase. Conversely, reducing environmental exposure of seabirds to debris, by reducing oceanic debris inputs or removing extant debris, should reduce the incidence of debris ingestion in seabirds.

In summary, there is now strengthened support that the seabird species at greatest risk of debris ingestion can be forecasted by examining their ecology, updated with the data presented on 51 Procellariiform species. Among the ecological drivers of variability in Procellariiform debris ingestion, the combination of taxonomy, foraging method, diet, and exposure to marine debris pollution are the most central factors driving incidence of marine debris ingestion. Expanding on our empirical results and using species distributions and marine debris density estimates in the ocean, we forecast that the species most at risk of ingesting debris forage at the surface by surface seizing, diving and filtering, have a crustacean dominant diet, and feed in or near marine debris hotspots.

## Methods

**Figure 5:**
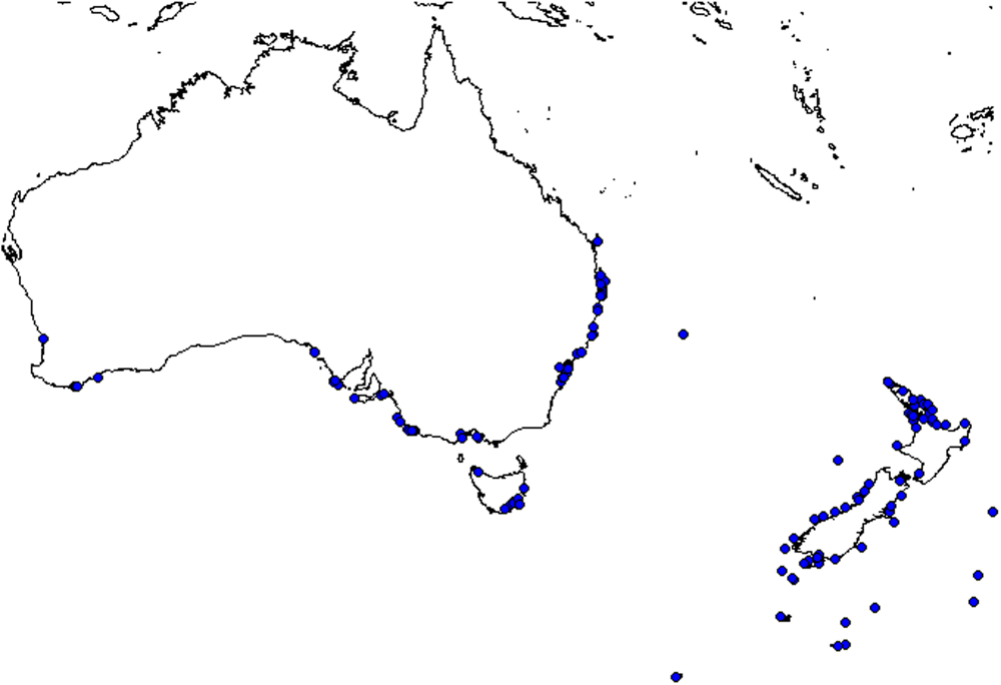
Locations of seabird carcass collection across Australia and New Zealand, including approximate oceanic by-catch locations.

Whole dead seabirds were obtained from fisheries by-catch, veterinary casualties, museum specimens and beach-washed carcasses from Australia and New Zealand between February 2013 and February 2017. Collections of deceased birds spanned from Perth, Western Australia in the West to Chatham Rise, off New Zealand in the east and from Fraser Is in the north to Macquarie in the south (Fig. 5). All methods were carried out in accordance with relevant guidelines and regulations

The birds were necropsied according to well-established collection and dissection procedures^30^. The age was recorded, and contents of the proventriculus and gizzard were removed and carefully visually inspected for anthropogenic debris (items of a natural origin such as pumice, squid beak, fish bone, shell, insect, seed and wood were excluded from analysis). All debris items visible to the naked eye were removed, rinsed in distilled water, and air dried before being stored in aluminium foil for counting and sorting.

Each seabird species was identified to species level and then categorized by genus, foraging range overlap with marine debris hotspots, presence or absence of constricted juncture between the proventriculus and gizzard (absent in albatross and present in other Procellariiformes^14,26^), primary diet and primary foraging behaviors. Diet categories included cephalopods, fish, crustaceans or scavenging. Foraging categories were surface diving, surface seizing, surface plunging, surface filtering, pursuit diving, pursuit plunging, pattering, hydroplaning, and dipping. Each diet and foraging method was recorded as “major importance”, “minor importance”, and “absent or rare”, for each species following Marchant and Higgins^22^. We used the R (Version 3.3.3)^31^ package “Rmixmod” cluster analysis to group species into diet clusters and foraging clusters, choosing the best number of clusters based on maximizing the log likelihood of the model. Marine debris hotspots followed Wilcox *et al*.^6^, and a diet cluster and foraging cluster was assigned to each species. Where range, diet and/or foraging behaviour were unknown, these species were excluded from associated analysis (SI Table 1).

Seabird species distribution and expected habitat use data were sourced from BirdLife International’s seabird database^32^, modified following Wilcox *et al*.^6^, and the debris density was determined following Wilcox *et al*.^6^. In brief, the ‘encounter density’ rate at which a seabird was likely to encounter debris within its foraging range was determined by multiplying the seabirds’ expected habitat use with the debris density in that location. Each species was allocated an ‘encounter density’ value, which was the sum of the debris density multiplied by the species’ expected use (the weighted distance from the edge of the range) of each 1° latitude by 1° longitude grid across its foraging range. Encounter density represents how many debris items a species would be expected to encounter during its average foraging activities.

Statistical analyses were performed using R (Version 3.3.3)^31^. We used generalized additive models (GAMs) to determine whether the sum of ingested debris items was significantly influenced by any of the factors described above. To determine the best model to explain the variation in the number of marine debris items ingested by a seabird, we used the “dredge” function in the “MuMIn” package to compare all possible factors, excluding species to prevent overfitting, and chose the model with the lowest Akaike information criterion (AIC). With this best model we tested interactions between ecological factors to obtain the best model to use for predicting marine debris ingestion in seabirds.

To support our analysis, we used three case studies of species with a known geographical foraging range. The first case study (1) examines two species, fluttering shearwater and fairy prion, which inhabit an overlapping range in the Tasman sea and share a similar diet but dissimilar foraging method to demonstrate the influence of foraging method. The second case study (2) examines the ranges of two geographically separated populations of flesh-footed shearwaters, *Ardenna carneipes*, supported by tracking data^27,28^, are used as a case study of drivers of variation in debris ingestion within a species. A third case study (3) examines a species that follows a known migration route, short-tailed shearwater and provides evidence of drivers of variation in debris ingestion across geography within a taxonomic group.

## Supplementary information

Supplementary information
