# Supplementary material for: Ecological drivers of marine debris ingestion in Procellariiform Seabirds
Source: Sci Rep. 2019 Jan 29;9:916. doi: 10.1038/s41598-018-37324-w (PMC6351657; doi:10.1038/s41598-018-37324-w)
Supplement: Supplementary file 1 — Supplementary information [file 41598_2018_37324_MOESM1_ESM.pdf]

Supplementary information

Ecological drivers of marine debris ingestion in Procellariiforme Seabirds

Lauren Roman[1*], Elizabeth Bell[2], Chris Wilcox[3], Britta Denise Hardesty[3], Mark Hindell[1, 4]

1 Institute for Marine and Antarctic Studies, University of Tasmania, Hobart, Tasmania, Australia,

2 Wildlife Management International, Blenheim, New Zealand,

3 Oceans and Atmosphere Flagship, CSIRO, Hobart, Tasmania, Australia,

4. Antarctic Climate and Ecosystems CRC, University of Tasmania, Hobart, Tasmania, Australia,

* lauren.roman@utas.edu.au

Table 1: Incidence of plastic ingestion in Australasian seabird species N is the number of individuals that were examined for debris ingestion. Percept debris ingestion is the percentage of the individuals sampled that had ingested debris. Median, standard deviation and maximum number of debris items ingested for each species. Diet clusters included Group 1: diet squid dominant, with some fish, crustaceans and scavenging. Group 2: diet fish dominant, but some squid and crustaceans. Group 3: diet crustacean dominant but may take fish and squid. Group 4: diet cephalopods and crustaceans dominant, with some also taking fish. Group 5: diet mostly fish and crustaceans. Group 6: diet mostly fish and squid with some scavenging. Foraging clusters included Group 1: mostly surface diving and pursuit diving/plunging. Group 2: mixed surface foraging including filtering, also seizing, plunging and dipping. Group 3: feeding under the surface by pursuit diving/plunging. Group 4: surface seizing and diving. Group 5: mostly pursuit plunging with some surface and pursuit diving. Group 6: surface seizing and plunging with minimal other feeding methods. Group 7: surface seizing, pattering and dipping. Encounter density is the sum of the oceanic debris density across the birds range as per Wilcox (2015), multiplied by the species' expected use of each area as per BirdLife international's seabird database [32].

Species	n	Percent debris ingestion	Median	Standard deviation	Max	Diet cluster	Foraging cluster	Encounter density
Diomedeidae- Albatrosses								
**Albatrosses**	263	0.02	0	0.3	4			
Antipodean albatross	1	0	0	NA	0	6	6	11.09
Black-browed albatross	9	11.1	0	0.3	1	2	6	155.9
Buller's albatross	90	1.1	0	0.2	2	1	6	5.77
Campbell albatross	4	0	0	0	0	2	6	16.24
Chatham Island albatross	1	0	0	NA	0	6	6	90.48
Gibson's albatross	2	0	0	0	0	6	6	11.09
Grey-headed albatross	4	25	0	2	4	6	6	26.25
Light-mantled sooty albatross	6	16.7	0	0.4	1	1	6	1.8
New Zealand white-capped albatross	85	0	0	0	0	6	6	141.15
Salvin's albatross	30	0	0	0	0	6	6	59.96
Shy albatross	26	3.8	0	0.2	1	6	6	141.15
Sooty albatross	1	0	0	NA	0	4	6	125.68
Southern royal albatross	2	0	0	0	0	1	6	11.56
Wandering albatross	5	0	0	0	0	6	6	145.19
Procellariidae- Petrels, Shearwaters and Prions								
**Procellarine petrels**	252	0.02	0	0.2	2			
Black petrel	7	0	0	0	0	2	6	9
Grey petrel	7	0	0	0	0	4	6	60.02
Tahiti petrel	1	0	0	NA	0	NA	NA	13
Westland petrel	16	12.5	0	0.5	2	2	4	2.97
White-chinned petrel	221	0.9	0	0.1	1	2	4	95.36
**Fulmarine petrels**	12	58.3	2	4.6	16			
Antarctic petrel	1	100	2	NA	2	4	6	5.6
Cape petrel	7	42.9	0	5.9	16	4	2	167.44
Southern fulmar	4	75	3	2.9	7	4	6	87.65
**Giant petrels**	12	58.3	1	7.5	25			

Northern giant petrel	4	50	0.5	2.4	5	1	6	87.03
Southern giant petrel	8	62.5	0	9.2	25	1	6	73.9
**Gadfly petrels**	37	19	0	0.53	2			
Black-winged petrel	2	0	0	0	0	3	7	108.8
Cook's petrel	7	14.3	0	0.76	2	1	NA	223.78
Gould's petrel	5	60	0.5	1	2	NA	NA	23.06
Great-winged petrel	4	0	0	0	0	1	7	86.68
Grey-faced petrel	4	0	0	0	0	1	7	86.68
Kerguelen petrel	3	33.3	0	0.58	1	4	6	37.71
Mottled petrel	3	33.3	0	0.6	1	1	6	243.38
Petrel (unknown sp)	1	0	0	NA	0	NA	NA	NA
Providence petrel	1	0	0	NA	0	NA	NA	155.38
White-headed petrel	6	16.7	0	1	NA	4	6	19.08
White-necked petrel	1	0	0	NA	0	6	7	101.93
**Shearwaters**	744	50.3	0	4.5	40			
Flesh-footed shearwater	213	25.8	0	3.2	36	6	5	398.14
Fluttering shearwater	70	10	0	0.5	3	5	3	15.38
Hutton's shearwater	4	0	0	0	0	5	3	8.75
Little shearwater	8	50	0.5	1.4	3	NA	NA	139.9
Short-tailed shearwater	332	86.4	4	4.8	27	4	1	292.2
Sooty shearwater	89	21.3	0	5.3	40	4	1	380.58
Wedge-tailed shearwater	28	14.3	0	1.7	9	2	6	120.02
**Prions**	372	38.4	0	3.1	30			
Antarctic prion	17	70.6	1.5	4.8	19	3	2	85.2
Blue petrel	2	50	15	21.2	30	4	7	37.56
Broad-billed prion	14	21.4	0	1.5	5	4	2	167.35
Fairy prion	236	25.8	0	1.6	14	5	7	46.26
Salvin's prion	24	70.8	2	3.3	14	3	2	NA
Slender-billed prion	79	60.8	1	3.6	22	3	7	28.84
Hydrobatidae – Storm petrels								
**Storm petrels**	7	85.7	13	8.11	23			

Black-bellied storm petrel	1	0	0	NA	0	6	7	503.68
White-faced storm petrel	6	100	13	7.1	23	5	7	290.7
Pelecanoididae- Diving petrels								
**Diving petrels**	32	6.3	0	0.4	2			
Common diving petrel	31	6.5	0	0.4	2	5	3	18.62
South Georgian diving petrel	1	0	0	NA	0	1	3	0.30

Table 2: The best model of which ecological variables can be used to explain how much marine debris is ingested by seabirds includes taxonomic grouping, foraging grouping, diet grouping and density of marine debris encountered in the foraging range. The model explains 48.9% of the variance in marine debris ingested by seabirds in our study.

Parametric coefficients:

<table>
<tr><th>Ecological Variables</th><th>Estimate</th><th>Std. Error</th><th>t value</th><th>Pr(>|t|)</th><th></th></tr>
<tr><td>Foraging 7 (Intercept)</td><td>7.308e-01</td><td>1.962e+00</td><td>0.372</td><td>0.7096</td><td></td></tr>
<tr><td>Foraging 6</td><td>-1.615e+00</td><td>9.311e-01</td><td>-1.734</td><td>0.0831</td><td>.</td></tr>
<tr><td>Foraging 5</td><td>4.091e+00</td><td>1.773e+00</td><td>2.307</td><td>0.0212</td><td>*</td></tr>
<tr><td>Foraging 4</td><td>4.204e+01</td><td>2.438e+05</td><td>0.000</td><td>0.9999</td><td></td></tr>
<tr><td>Foraging 3</td><td>-1.177e+00</td><td>1.992e+00</td><td>-0.591</td><td>0.5548</td><td></td></tr>
<tr><td>Foraging 2</td><td>-6.869e-01</td><td>7.661e-01</td><td>-0.897</td><td>0.3701</td><td></td></tr>
<tr><td>Foraging 1</td><td>3.427e+00</td><td>2.225e+00</td><td>1.540</td><td>0.1237</td><td></td></tr>
<tr><td>Storm petrels</td><td>7.308e+00</td><td>2.253e+00</td><td>3.244</td><td>0.0012</td><td>**</td></tr>
<tr><td>Prions</td><td>1.144e+00</td><td>2.183e+00</td><td>0.524</td><td>0.6002</td><td></td></tr>
<tr><td>Shearwaters</td><td>3.851e-01</td><td>8.951e-01</td><td>0.430</td><td>0.6671</td><td></td></tr>
<tr><td>Gadfly petrels</td><td>-9.351e-01</td><td>2.141e+00</td><td>-0.437</td><td>0.6623</td><td></td></tr>
<tr><td>Fulmarine petrels</td><td>2.203e+00</td><td>2.068e+00</td><td>1.065</td><td>0.2869</td><td></td></tr>
<tr><td>Procellarine petrels</td><td>-4.723e+01</td><td>2.438e+05</td><td>0.000</td><td>0.9998</td><td></td></tr>
<tr><td>Giant petrels</td><td>3.936e+00</td><td>1.690e+00</td><td>2.329</td><td>0.0200</td><td>*</td></tr>
<tr><td>Albatrosses</td><td>-1.562e+00</td><td>1.571e+00</td><td>-0.994</td><td>0.3202</td><td></td></tr>
<tr><td>Diet 5</td><td>-1.729e+00</td><td>1.025e+00</td><td>-1.687</td><td>0.0919</td><td>.</td></tr>
<tr><td>Diet 4</td><td>8.874e-01</td><td>1.144e+00</td><td>0.776</td><td>0.4379</td><td></td></tr>
<tr><td>Diet 3</td><td>-5.698e-01</td><td>1.053e+00</td><td>-0.541</td><td>0.5887</td><td></td></tr>
<tr><td>Diet 2</td><td>1.309e+00</td><td>1.347e+00</td><td>0.971</td><td>0.3315</td><td></td></tr>
<tr><td>Diet 1</td><td>-5.997e-01</td><td>8.164e-01</td><td>-0.735</td><td>0.4627</td><td></td></tr>
<tr><td>Encounter density</td><td>-1.314e-02</td><td>1.941e-03</td><td>-6.769</td><td>1.81e-11</td><td>***</td></tr>
</table>

Signif. codes: 0 ‘*’ 0.001 ‘**’ 0.01 ‘*’ 0.05 ‘.’ 0.1 ‘ ’ 1**
